# Association of Race With Receipt of Proton Beam Therapy for Patients With Newly Diagnosed Cancer in the US, 2004-2018

**DOI:** 10.1001/jamanetworkopen.2022.8970

**Published:** 2022-04-26

**Authors:** Leticia M. Nogueira, Helmneh M. Sineshaw, Ahmedin Jemal, Craig E. Pollack, Jason A. Efstathiou, K. Robin Yabroff

**Affiliations:** 1 Department of Surveillance and Health Equity Science, American Cancer Society, Atlanta, Georgia; 2 Department of Health Policy and Management, Johns Hopkins Bloomberg School of Public Health and Johns Hopkins School of Nursing, Baltimore, Maryland; 3 Department of Radiation Oncology, Massachusetts General Hospital, Boston

## Abstract

**Importance:**

Black patients are less likely than White patients to receive guideline-concordant cancer care in the US. Proton beam therapy (PBT) is a potentially superior technology to photon radiotherapy for tumors with complex anatomy, tumors surrounded by sensitive tissues, and childhood cancers.

**Objective:**

To evaluate whether there are racial disparities in the receipt of PBT among Black and White individuals diagnosed with all PBT-eligible cancers in the US.

**Design, Setting, and Participants:**

This cross-sectional study evaluated Black and White individuals diagnosed with PBT-eligible cancers between January 1, 2004, and December 31, 2018, in the National Cancer Database, a nationwide hospital-based cancer registry that collects data on radiation treatment, even when it is received outside the reporting facility. American Society of Radiation Oncology model policies were used to classify patients into those for whom PBT is the recommended radiation therapy modality (group 1) and those for whom evidence of PBT efficacy is still under investigation (group 2). Propensity score matching was used to ensure comparability of Black and White patients’ clinical characteristics and regional availability of PBT according to the National Academy of Medicine’s definition of disparities. Data analysis was performed from October 4, 2021, to February 22, 2022.

**Exposure:**

Patients’ self-identified race was ascertained from medical records.

**Main Outcomes and Measures:**

The main outcome was receipt of PBT, with disparities in this therapy’s use evaluated with logistic regression analysis.

**Results:**

Of the 5 225 929 patients who were eligible to receive PBT and included in the study, 13.6% were Black, 86.4% were White, and 54.3% were female. The mean (SD) age at diagnosis was 63.2 (12.4) years. Black patients were less likely to be treated with PBT than their White counterparts (0.3% vs 0.5%; odds ratio [OR], 0.67; 95% CI, 0.64-0.71). Racial disparities were greater for group 1 cancers (0.4% vs 0.8%; OR, 0.49; 95% CI, 0.44-0.55) than group 2 cancers (0.3% vs 0.4%; OR, 0.75; 95% CI, 0.70-0.80). Racial disparities in PBT receipt among group 1 cancers increased over time (annual percent change = 0.09, *P* < .001) and were greatest in 2018, the most recent year of available data.

**Conclusions and Relevance:**

In this cross-sectional study, Black patients were less likely to receive PBT than their White counterparts, and disparities were greatest for cancers for which PBT was the recommended radiation therapy modality. These findings suggest that efforts other than increasing the number of facilities that provide PBT will be needed to eliminate disparities.

## Key Points

**Question:**

Are there racial disparities in the use of proton beam therapy (PBT) among individuals newly diagnosed with cancer in the US?

**Findings:**

In this cross-sectional study, Black patients were less likely to be treated with PBT than White patients, especially for cancers for which PBT is recommended over photon-based radiation therapy. The racial disparities in receipt of PBT increased over time despite increases in the number of facilities offering PBT in the US.

**Meaning:**

These findings indicate that racial disparities in the use of PBT are substantial, especially for cancers for which PBT is the recommended radiation therapy modality, and that increased PBT availability did not help eliminate racial disparities for these cancers.

## Teaser

This cross-sectional study of Black and White patients with cancer assesses racial disparities in the receipt of proton beam therapy.

## Introduction

Proton beam therapy (PBT) is potentially superior to photon radiation therapy (RT) for tumors with complex anatomy surrounded by sensitive tissues and for childhood cancers, for which decreasing late effects of RT is a major concern.^1,2^ Black patients are less likely to receive any RT,^3^ including use of advanced technologies.^4,5,6^ Previous studies investigating disparities in receipt of PBT evaluated only a single cancer site,^7,8,9,10,11,12,13,14,15^ age group,^16^ or geographic region^17^ and, importantly, only included patients receiving RT rather than all patients for whom RT is recommended. Because Black patients are less likely than White patients to receive any type of RT,^3,6,18,19,20,21,22,23,24^ these studies^7,10,11,12,13,16,25,26,27,28,29,30^ might have underestimated the racial disparity in receipt of PBT. Furthermore, differences in referral patterns and regional availability of cancer therapy modalities can influence cancer care receipt^31,32,33,34,35,36^ and racial disparities in access to care.^37,38,39^ The aim of this study was to conduct a comprehensive evaluation of racial disparities in receipt of PBT among individuals diagnosed with all PBT-eligible cancers in the US using recent nationwide data.

## Methods

### Data Source and Study Cohort

The National Cancer Database (NCDB) is a nationwide hospital-based cancer registry jointly sponsored by the American College of Surgeons and the American Cancer Society that captures approximately 72% of all cancer cases in the US from more than 1500 facilities accredited by the American College of Surgeons’ Commission on Cancer and collects RT information even when it is received outside the reporting facility.^40,41^ Therefore, the NCDB captures PBT received both at NCDB facilities (59.5% of PBT patients in this study) and at facilities outside the NCDB (40.5% of PBT patients in this study). This cross-sectional study was granted exemption from review by the institutional review board of the Morehouse School of Medicine in Atlanta, Georgia because the study involves secondary data analysis only; therefore, informed consent was not required. All data were deidentified. This study followed the Strengthening the Reporting of Observational Studies in Epidemiology (STROBE) reporting guideline.

Because Black patients are less likely than White patients to receive any type of cancer treatment,^42^ including RT,^3,6,18,19,20,21,22,23,24^ the study sample was not restricted to patients who received RT to avoid biasing the disparity estimates toward the null. To account for PBT availability and patient opportunity for referral, only patients diagnosed or treated at facilities that reported at least 5 patients receiving PBT between January 1, 2004, and December 31, 2018, or patients who were treated by a radiation oncologist who treated at least 5 patients with PBT between January 1, 2004, and December 31, 2018 (n = 3 961 245), were included. The following patients were excluded from the analysis: patients with missing race information or with race or ethnicity other than non-Hispanic Black and non-Hispanic White (n = 765 912) and patients diagnosed with cancer sites for which fewer than 10 Black patients received PBT throughout the study period because of sparse data to generate stable estimates (n = 1 138 057).

### Measures

We used the American Society of Radiation Oncology (ASTRO) model policies published in 2017 to classify patients into group 1 and group 2 according to cancer type and RT anatomical target.^43^ Group 1 (those for whom PBT is the recommended radiation therapy modality) included patients diagnosed with ocular, head and neck (mouth, parotid gland, tonsil, oropharynx, nasopharynx, pyriform sinus, hypopharynx, and paranasal sinuses), central nervous system (CNS) (including cerebral meninges, brain, spinal cord, and other CNS), hepatocellular, skull and spine, and rhabdomyosarcoma cancers. Group 2 (those for whom evidence of PBT efficacy is still under investigation) included patients diagnosed with cancers of prostate, lung, breast, esophagus, colorectum, anus, uterus, cervix, and pancreas and Hodgkin lymphoma.

Patients’ self-identified race was ascertained from medical records. Patient comorbidities were identified according to the modified Charlson-Deyo Comorbidity Index for patients with cancer and categorized into 0, 1, or 2 or greater.^44^

We used propensity score matching to ensure that Black and White patients’ clinical characteristics and regional availability of PBT were comparable. We generated a propensity score for each patient that predicted the probability of being Black. Variables that were deemed relevant a priori using the National Academy of Medicine definition of disparity as “differences in health care services received by the two groups that are not due to underlying health care needs”^45,46^ were included in the propensity score model, and no further filtering or selection was conducted. Because structural racism is a primary cause of racial differences in socioeconomic status (SES) by limiting access to education, employment opportunities, and intergenerational transfer of wealth,^47^ we chose not to match on SES variables when estimating racial disparities in receipt of PBT. Similarly, because the Social Security Act of 1935 created a system of employment-based health insurance coverage that interacts with discriminatory hiring practices^48^ to restrict access to health care for racialized groups, we chose not to match on health insurance coverage when estimating racial disparities in receipt of PBT. For each cancer site, the propensity score model included age at diagnosis, sex, cancer stage, comorbidities, year of diagnosis, and geographic region. Patients were matched (1:1) on propensity scores using a greedy match,^49^ wherein Black patients were matched to the nearest White patient, starting with the best match.^50^ To estimate the contribution of modifiable factors to the racial disparity in receipt of PBT,^42^ patients’ zip code of residence median income quintile and health insurance coverage type were further added to separate propensity score models in addition to matching on PBT eligibility and availability.

### Statistical Analysis

Data analyses were conducted from October 4, 2021, to February 22, 2022. We used the standardized difference to compare the balance between variables,^51^ with no imbalance after propensity score matching (eTables 1-3 in the Supplement). Therefore, unadjusted odds ratios (ORs) and 95% CIs are presented.^52^ We used χ^2^ statistics to compare patients’ characteristics and logistic regression to compare disparities in receipt of PBT by racialized group. To characterize trends in racial disparities in PBT use through time, annual percent change (APC) of the absolute difference in PBT receipt between Black and White patients was calculated by fitting a least-squares regression using year of diagnosis as the independent variable. Changes in trends (structural breaks) were identified by using the additive outliers method.^53^ In sensitivity analysis, we excluded stage IV cancers, for which PBT is often not recommended as first-course treatment, and stratified the breast cancer analysis by laterality. All analyses were performed using SAS software, version 9.4 (SAS Institute Inc). Statistical significance was set at 2-sided α = .05.

## Results

Of the 5 225 929 patients eligible for PBT and included the study, 4 515 679 (86.4%) were White and 710 250 (13.6%) were Black; 2 837 066 (54.3%) were female and 2 388 863 (45.7%) were male; and mean (SD) age at diagnosis was 63.2 (12.4) years. At baseline, Black patients were younger and more likely to be diagnosed with hepatocellular, prostate, and cervical cancers; be uninsured or covered by Medicaid; have comorbidities; be treated at teaching hospitals; live in lower-income and metropolitan areas; and be diagnosed more recently. White patients were more likely to be diagnosed with stage I cancer (Table 1). Less than 1% of patients received PBT for most cancer sites included in the study (eTable 4 in the Supplement).

**Table 1.** Characteristics of Black and White Patients Diagnosed With PBT-Eligible Cancers at Baseline and at Each Propensity Score Matching Step, National Cancer Database (2004-2018)^a^

Characteristic	Baseline		*P* value	Eligibility and availability match^b^		*P* value	Insurance match		*P* value	Income match		*P* value
	Black	White		Black	White		Black	White		Black	White	
Total	710 250 (100)	4 515 679 (100)	NA	679 883 (100)	679 883 (100)	NA	683 976 (100)	683 976 (100)	NA	682 489 (100)	682 489 (100)	NA
Age, mean (SD), y	60.5 (13.2)	63.6 (13.4)	NA	60.5 (13.3)	60.5 (13.3)	NA	60.7 (13.2)	6.7 (13.1)	NA	60.8 (13.1)	60.8 (13.1)	NA
Age group												
Children (<15 y)	3960 (0.6)	19 288 (0.4)	<.001	3727 (0.5)	3672 (0.5)	>.99	3760 (0.5)	3725 (.5)	.40	3832 (0.6)	3726 (0.5)	.10
AYA (15-39 y)	36 468 (5.1)	167 310 (3.7)		35 225 (5.2)	35 097 (5.2)		33 486 (4.9)	33 048 (4.8)		32 727 (4.8)	31 904 (4.7)	
Adult (40-64 y)	392 122 (55.2)	2 074 113 (45.9)		375 370 (55.2)	375 650 (55.3)		375 790 (54.9)	376 479 (55.0)		374 706 (54.9)	375 008 (54.9)	
Older adult (65-74 y)	181 754 (25.6)	1 320 970 (29.3)		172 188 (25.3)	172 311 (25.3)		177 180 (25.9)	177 549 (26.0)		177 615 (26.0)	178 947 (26.2)	
Elderly (≥75 y)	95 946 (13.5)	933 998 (20.7)		93 373 (13.7)	93 153 (13.7)		93 760 (13.7)	93 175 (13.6)		93 609 (13.7)	92 904 (13.6)	
Sex												
Male	330 083 (46.5)	2 058 780 (45.6)	<.001	311 377 (45.8)	312 430 (46.0)	>.99	317 361 (46.4)	317 997 (46.5)	.50	318 480 (46.7)	320 366 (46.9)	.20
Female	380 167 (53.5)	2 456 899 (54.4)		368 506 (54.2)	367 453 (54.0)		366 615 (53.6)	365 979 (53.5)		364 009 (53.3)	362 123 (53.1)	
PBT group												
1	121 082 (17.0)	776 168 (17.2)	<.001	117 364 (17.3)	117 364 (17.3)	>.99	117 623 (17.2)	117 623 (17.2)	>.99	119 739 (17.5)	119 739 (17.5)	>.99
2	589 168 (83.0)	3 739 511 (82.8)		562 519 (82.7)	562 519 (82.7)		566 353 (82.8)	566 353 (82.8)		562 750 (82.5)	562 750 (82.5)	
**Cancer site**													
Group 1												
Head and neck	31 214 (4.4)	223 440 (4.9)	<.001	29 069 (4.3)	29 069 (4.3)	>.99	30 314 (4.4)	30 314 (4.4)	>.99	30 894 (4.5)	30 894 (4.5)	>.99
CNS	61 104 (8.6)	402 805 (8.9)		60 816 (8.9)	60 816 (8.9)		59 397 (8.7)	59 397 (8.7)		60 575 (8.9)	60 575 (8.9)	
Hepatocellular	27 040 (3.8)	120 499 (2.7)		25 969 (3.8)	25 969 (3.8)		26 310 (3.8)	26 310 (3.8)		26 652 (3.9)	26 652 (3.9)	
Skull and spine	455 (0.1)	4742 (0.1)		444 (0.1)	444 (0.1)		416 (0.1)	416 (.1)		432 (0.1)	432 (0.1)	
Ocular	662 (0.1)	22 341 (0.5)		535 (0.1)	535 (0.1)		623 (0.1)	623 (.1)		626 (0.1)	626 (0.1)	
Rhabdomyosarcoma	607 (0.1)	2341 (0.1)		531 (0.1)	531 (0.1)		563 (0.1)	563 (0.1)		560 (0.1)	560 (0.1)	
Group 2												
Prostate	171 545 (24.2)	907 878 (20.1)		170 435 (25.1)	170 435 (25.1)		163 435 (23.9)	163 435 (23.9)		163 685 (24.0)	163 685 (24.0)	
Lung	112 112 (15.8)	830 771 (18.4)		91 479 (13.5)	91 479 (13.5)		109 810 (16.1)	109 810 (16.1)		111 568 (16.3)	111 568 (16.3)	
Breast	225 236 (31.7)	1 462 605 (32.4)		222 036 (32.7)	222 036 (32.7)		215 114 (31.5)	215 114 (31.5)		212 936 (31.2)	212 936 (31.2)	
Colon and rectum	27 466 (3.9)	209 633 (4.6)		26 531 (3.9)	26 531 (3.9)		26 804 (3.9)	26 804 (3.9)		25 597 (3.8)	25 597 (3.8)	
Anal	3163 (0.4)	20 756 (0.5)		2988 (0.4)	2988 (0.4)		2951 (0.4)	2951 (.4)		2235 (0.3)	2235 (0.3)	
Uterus	10 619 (1.5)	48 544 (1.1)		10 547 (1.6)	10 547 (1.6)		10 331 (1.5)	10 331 (1.5)		10 285 (1.5)	10 285 (1.5)	
Cervix	10 561 (1.5)	40 482 (0.9)		10 479 (1.5)	10 479 (1.5)		10 251 (1.5)	10 251 (1.5)		9315 (1.4)	9315 (1.4)	
Pancreas	15 762 (2.2)	105 734 (2.3)		15 540 (2.3)	15 540 (2.3)		15 402 (2.3)	15 402 (2.3)		14 760 (2.2)	14 760 (2.2)	
Esophagus	7244 (1.0)	80 210 (1.8)		7185 (1.1)	7185 (1.1)		7027 (1.0)	7027 (1.0)		7094 (1.0)	7094 (1.0)	
Hodgkin lymphoma	5460 (0.8)	32 898 (0.7)		5299 (0.8)	5299 (0.8)		5228 (0.8)	5228 (.8)		5275 (0.8)	5275 (0.8)	
Stage												
0	44 823 (6.3)	258 977 (5.7)	<.001	44 245 (6.5)	44 134 (6.5)	>.99	42 988 (6.3)	42 871 (6.3)	.12	42 344 (6.2)	42 272 (6.2)	.40
I	159 038 (22.4)	1 280 313 (28.4)		153 953 (22.6)	154 489 (22.7)		154 946 (22.7)	155 240 (22.7)		155 168 (22.7)	157 033 (23.0)	
II	210 350 (29.6)	1 202 031 (26.6)		208 152 (30.6)	207 802 (30.6)		201 872 (29.5)	201 604 (29.5)		201 038 (29.5)	200 555 (29.4)	
III	107 565 (15.1)	629 522 (13.9)		99 382 (14.6)	99 968 (14.7)		103 299 (15.1)	103 282 (15.1)		102 162 (15.0)	101 816 (14.9)	
IV	96 524 (13.6)	552 129 (12.2)		83 288 (12.3)	83 657 (12.3)		92 497 (13.5)	93 500 (13.7)		92 519 (13.6)	92 599 (13.6)	
Unknown	91 950 (12.9)	592 707 (13.1)		90 863 (13.4)	89 833 (13.2)		88 374 (12.9)	87 479 (12.8)		89 258 (13.1)	88 214 (12.9)	
Region												
Northeast	127 022 (18.0)	975 492 (21.6)	<.001	126 189 (18.6)	124 438 (18.3)	>.99	123 012 (18.0)	120 188 (17.6)	>.99	120 988 (17.7)	113 609 (16.6)	>.99
Midwest	131 403 (18.6)	1 188 469 (26.4)		130 136 (19.1)	129 276 (19.0)		128 702 (18.8)	127 403 (18.6)		126 222 (18.5)	121 317 (17.8)	
South	410 885 (58.1)	1 643 872 (36.5)		385 337 (56.7)	388 454 (57.1)		394 307 (57.6)	399 278 (58.4)		397 345 (58.2)	410 108 (60.1)	
West	38 279 (5.4)	699 843 (15.5)		38 221 (5.6)	37 715 (5.5)		37 955 (5.5)	37 107 (5.4)		37 934 (5.6)	37 455 (5.5)	
Insurance												
Private	296 445 (42.6)	2 073 764 (46.7)	<.001	286 756 (43.0)	355 617 (54.0)	<.001	294 029 (43.0)	297 864 (43.5)	.10	283 939 (42.4)	331 207 (49.8)	<.0001
Uninsured	33 710 (4.8)	83 610 (1.9)		30 921 (4.6)	15 911 (2.4)		31 497 (4.6)	30 843 (4.5)		31 791 (4.8)	19 760 (3.0)	
Medicaid	86 117 (12.4)	186 911 (4.2)		82 014 (12.3)	32 295 (4.9)		80 336 (11.7)	78 664 (11.5)		81 115 (12.1)	42 292 (6.4)	
Medicare	271 030 (38.9)	2 064 264 (46.5)		258 635 (38.8)	250 012 (38.0)		270 016 (39.5)	268 846 (39.3)		263 700 (39.4)	265 971 (40.0)	
Other	8630 (1.2)	30 624 (0.7)		8080 (1.2)	4808 (0.7)		8098 (1.2)	7759 (1.1)		8424 (1.3)	5992 (0.9)	
Facility type												
NCI designated	128 719 (18.6)	860 277 (19.3)	<.001	123 966 (18.7)	340 291 (52.3)	<.001	121 867 (18.2)	288 407 (43.8)	<.001	123 516 (18.5)	230 644 (34.7)	<.001
Comprehensive	195 788 (28.2)	1 622 477 (36.4)		186 281 (28.1)	220 868 (34.0)		189 743 (28.4)	236 544 (35.9)		189 527 (28.4)	259 922 (39.1)	
Teaching	227 259 (32.8)	1 019 750 (22.8)		217 337 (32.8)	55 909 (8.6)		218 553 (32.7)	83 599 (12.7)		217 168 (32.6)	104 924 (15.8)	
Community	5878 (0.8)	75 800 (1.7)		5530 (0.8)	3042 (0.5)		5697 (0.9)	4753 (0.7)		5630 (0.8)	7023 (1.1)	
Other	135 545 (19.6)	884 837 (19.8)		130 407 (19.7)	30 068 (4.6)		132 244 (19.8)	45 634 (6.9)		130 600 (19.6)	61 799 (9.3)	
Comorbidity												
0	517 296 (72.8)	3 466 053 (76.8)	<.001	493 933 (72.6)	499 774 (73.5)	.10	497 310 (72.7)	500 049 (73.1)	.10	499 062 (73.1)	505 431 (74.1)	.10
1	129 192 (18.2)	726 601 (16.1)		124 749 (18.3)	122 785 (18.1)		125 214 (18.3)	124 493 (18.2)		123 324 (18.1)	120 689 (17.7)	
≥2	63 762 (9.0)	323 025 (7.2)		61 201 (9.0)	57 324 (8.4)		61 452 (9.0)	59 434 (8.7)		60 103 (8.8)	56 369 (8.3)	
Area												
Metropolitan	640 144 (92.6)	3 671 453 (84.7)	<.001	616 680 (92.8)	535 496 (83.6)	<.001	619 364 (92.6)	538 141 (82.8)	<.001	619 339 (92.6)	491 823 (75.6)	<.001
Urban	45 211 (6.5)	588 600 (13.6)		41 988 (6.3)	93 862 (14.6)		43 438 (6.5)	99 361 (15.3)		43 347 (6.5)	137 810 (21.2)	
Rural	6084 (0.9)	75 590 (1.7)		5616 (0.8)	11 436 (1.8)		5851 (0.9)	12 751 (2.0)		5817 (0.9)	20 684 (3.2)	
Income, $												
<36 000	248 415 (35.3)	399 409 (8.9)	<.001	71 566 (10.6)	236 840 (35.0)	<.001	239 567 (35.2)	81 407 (12.0)	<.001	229 278 (33.6)	225 212 (33.0)	.10
36 000-43 999	132 959 (18.9)	700 762 (15.6)		114 927 (17.0)	127 498 (18.8)		128 306 (18.8)	122 784 (18.0)		131 294 (19.2)	135 295 (19.8)	
44 000-52 999	108 942 (15.5)	859 123 (19.1)		131 220 (19.4)	104 575 (15.4)		105 424 (15.5)	135 247 (19.9)		108 253 (15.9)	110 745 (16.2)	
53 000-68 999	120 435 (17.1)	1 168 870 (26.1)		161 446 (23.9)	116 564 (17.2)		116 667 (17.1)	159 362 (23.4)		119 972 (17.6)	120 447 (17.6)	
69 000+	93 945 (13.3)	1 358 645 (30.3)		197 571 (29.2)	91 641 (13.5)		91 256 (13.4)	182 216 (26.8)		93 692 (13.7)	90 790 (13.3)	
Diagnosis year												
2004	33 107 (4.7)	244 085 (5.4)	<.001	31 915 (4.7)	31 872 (4.7)	.10	31 533 (4.6)	31 356 (4.6)	.10	31 462 (4.6)	32 100 (4.7)	.10
2005	34 807 (4.9)	250 490 (5.5)		33 464 (4.9)	33 500 (4.9)		33 270 (4.9)	33 195 (4.9)		33 158 (4.9)	33 307 (4.9)	
2006	37 584 (5.3)	266 409 (5.9)		36 326 (5.3)	36 361 (5.3)		36 055 (5.3)	36 024 (5.3)		36 079 (5.3)	36 146 (5.3)	
2007	40 732 (5.7)	279 873 (6.2)		39 430 (5.8)	39 436 (5.8)		39 312 (5.7)	39 092 (5.7)		39 112 (5.7)	38 719 (5.7)	
2008	42 883 (6.0)	286 278 (6.3)		41 253 (6.1)	41 090 (6.0)		41 381 (6.1)	41 229 (6.0)		41 255 (6.0)	40 940 (6.0)	
2009	45 992 (6.5)	293 947 (6.5)		44 162 (6.5)	44 140 (6.5)		44 285 (6.5)	44 178 (6.5)		44 197 (6.5)	43 868 (6.4)	
2010	46 566 (6.6)	290 994 (6.4)		44 751 (6.6)	44 633 (6.6)		44 772 (6.5)	44 582 (6.5)		44 807 (6.6)	43 958 (6.4)	
2011	48 541 (6.8)	303 307 (6.7)		46 691 (6.9)	46 753 (6.9)		46 783 (6.8)	46 670 (6.8)		46 830 (6.9)	46 411 (6.8)	
2012	49 302 (6.9)	301 376 (6.7)		47 272 (7.0)	47 226 (6.9)		47 431 (6.9)	47 179 (6.9)		47 580 (7.0)	46 813 (6.9)	
2013	51 431 (7.2)	312 390 (6.9)		48 985 (7.2)	48 983 (7.2)		49 534 (7.2)	49 575 (7.2)		49 643 (7.3)	49 018 (7.2)	
2014	52 560 (7.4)	318 410 (7.1)		50 036 (7.4)	49 992 (7.4)		50 719 (7.4)	50 736 (7.4)		50 603 (7.4)	50 391 (7.4)	
2015	54 042 (7.6)	331 844 (7.3)		51 414 (7.6)	51 355 (7.6)		51 920 (7.6)	52 602 (7.7)		52 086 (7.6)	52 686 (7.7)	
2016	55 380 (7.8)	337 730 (7.5)		52 678 (7.7)	52 949 (7.8)		53 561 (7.8)	54 098 (7.9)		53 204 (7.8)	54 100 (7.9)	
2017	57 997 (8.2)	349 075 (7.7)		55 005 (8.1)	55 161 (8.1)		56 159 (8.2)	56 606 (8.3)		55 715 (8.2)	56 860 (8.3)	
2018	59 326 (8.4)	349 471 (7.7)		56 501 (8.3)	56 432 (8.3)		57 261 (8.4)	56 854 (8.3)		56 758 (8.3)	57 172 (8.4)	

Abbreviations: AYA, adolescent and young adult; CNS, central nervous system; NA, not applicable; NCI, National Cancer Institute; PBT, proton beam therapy.

^a^ Data are presented as number (percentage) of patients unless otherwise indicated.

^b^ Eligibility and availability match for each cancer site included age at diagnosis, sex, cancer stage, comorbidities, year of diagnosis, and geographic region.

Black patients were significantly less likely (OR, 0.67; 95% CI, 0.64-0.71) to receive PBT overall and by each ASTRO indication group than their White counterparts (Table 2). Racial disparity in receipt of PBT was higher in group 1 cancers (0.4% vs 0.8%; OR, 0.49; 95% CI, 0.44-0.55) than in group 2 cancers (0.3% vs 0.4%; OR, 0.75; 95% CI, 0.70-0.80) and was statistically significant for rhabdomyosarcoma (OR, 0.50 95% CI, 0.34-0.75), CNS (OR, 0.46; 95% CI, 0.39-0.53), head and neck (OR, 0.48; 95% CI, 0.39-0.59), and hepatocellular (OR, 0.47; 95% CI, 0.28-0.78) cancer (group 1) and prostate (OR, 0.83; 95% CI, 0.76-0.91), breast (OR, 0.60; 95% CI, 0.52-0.68), lung (OR, 0.62; 95% CI, 0.51-0.75), and esophagus (OR, 0.57; 95% CI, 0.36-0.90) cancer (group 2).

**Table 2.** Receipt of PBT Among Black and White Patients Propensity Score Matched on PBT Eligibility and Availability and Then on Health Insurance Coverage Type or Income, National Cancer Database (2004-2018)

Group	PBT, No. (%)		OR (95% CI)^a^		
	No	Yes	Eligibility and availability	Insurance	Income
Overall					
Black	677 783 (99.7)	2100 (0.3)	0.67 (0.64-0.71)	0.72 (0.68-0.76)	0.73 (0.69-0.78)
White	676 760 (99.5)	3123 (0.5)			
**ASTRO indication group**					
Group 1					
Black	116 885 (99.6)	479 (0.4)	0.49 (0.44-0.55)	0.53 (0.47-0.59)	0.57 (0.51-0.64)
White	116 399 (99.2)	965 (0.8)			
Group 2					
Black	560 898 (99.7)	1621 (0.3)	0.75 (0.70-0.80)	0.81 (0.76-0.86)	0.80 (0.75-0.86)
White	560 361 (99.6)	2158 (0.4)			
**Cancer sites**					
Group 1					
Head and neck					
Black	28 940 (99.6)	129 (0.4)	0.48 (0.39-0.59)	0.51 (0.41-0.63)	0.59 (0.48-0.73)
White	28 802 (99.1)	267 (0.9)			
CNS					
Black	60 575 (99.6)	241 (0.4)	0.46 (0.39-0.53)	0.48 (0.41-0.57)	0.51 (0.43-0.59)
White	60 289 (99.1)	527 (0.9)			
Hepatocellular					
Black	25 948 (99.9)	21 (0.1)	0.47 (0.28-0.78)	0.47 (0.27-0.82)	0.64 (0.37-1.10)
White	25 924 (99.8)	45 (0.2)			
Skull and spine					
Black	409 (92.1)	35 (7.9)	0.89 (0.55-1.43)	0.89 (0.55-1.44)	0.89 (0.55-1.44)
White	405 (91.2)	39 (8.8)			
Ocular					
Black	523 (97.8)	12 (2.2)	1.09 (0.48-2.50)	1.38 (0.55-3.46)	2.02 (0.75-5.42)
White	524 (97.9)	11 (2.1)			
Rhabdomyosarcoma					
Black	490 (92.3)	41 (7.7)	0.50 (0.34-0.75)	0.63 (0.43-0.92)	0.57 (0.39-0.84)
White	455 (85.7)	76 (14.3)			
Group 2					
Prostate					
Black	169 533 (99.5)	902 (0.5)	0.83 (0.76-0.91)	0.85 (0.77-0.93)	0.80 (0.74-0.88)
White	169 349 (99.4)	1086 (0.6)			
Lung					
Black	91 302 (99.8)	177 (0.2)	0.62 (0.51-0.75)	0.74 (0.62-0.89)	0.84 (0.70-1.01)
White	91 194 (99.7)	285 (0.3)			
Breast					
Black	221 689 (99.8)	347 (0.2)	0.60 (0.52-0.68)	0.65 (0.57-0.75)	0.68 (0.59-0.78)
White	221 455 (99.7)	581 (0.3)			
Esophagus					
Black	7156 (99.6)	29 (0.4)	0.57 (0.36-0.90)	0.74 (0.46-1.20)	1.00 (0.60-1.67)
White	7134 (99.3)	51 (0.7)			
Hodgkin lymphoma					
Black	5262 (99.3)	37 (0.7)	0.80 (0.52-1.24)	1.23 (0.76-2.00)	0.88 (0.56-1.37)
White	5253 (99.1)	46 (0.9)			
Colorectal					
Black	26 494 (99.9)	37 (0.1)	1.06 (0.67-1.68)	1.03 (0.65-1.63)	1.00 (0.62-1.62)
White	26 496 (99.9)	35 (0.1)			
Anal					
Black	2968 (99.3)	20 (0.7)	1.05 (0.56-1.98)	1.31 (0.68-2.52)	1.90 (0.84-4.26)
White	2969 (99.4)	19 (0.6)			
Pancreas					
Black	15 509 (99.8)	31 (0.2)	1.03 (0.63-1.71)	1.07 (0.64-1.77)	1.80 (0.83-3.90)
White	15 510 (99.8)	30 (0.2)			
Cervix					
Black	10 456 (99.8)	23 (0.2)	2.30 (1.10-4.84)	1.92 (0.95-3.86)	1.55 (0.72-3.30)
White	10 469 (99.9)	10 (0.1)			
Uterus					
Black	10 529 (99.8)	18 (0.2)	1.20 (0.60-2.38)	1.39 (0.68-2.83)	1.80 (0.83-3.90)
White	10 532 (99.9)	15 (0.1)			

Abbreviations: ASTRO, American Society for Radiation Oncology; CNS, central nervous system; OR, odds ratio; PBT, proton beam therapy.

^a^ For each cancer site, Black and White patients were propensity score matched on age, sex, cancer stage at diagnosis, comorbidities, year of diagnosis, and geographic region.

**Figure 1.**
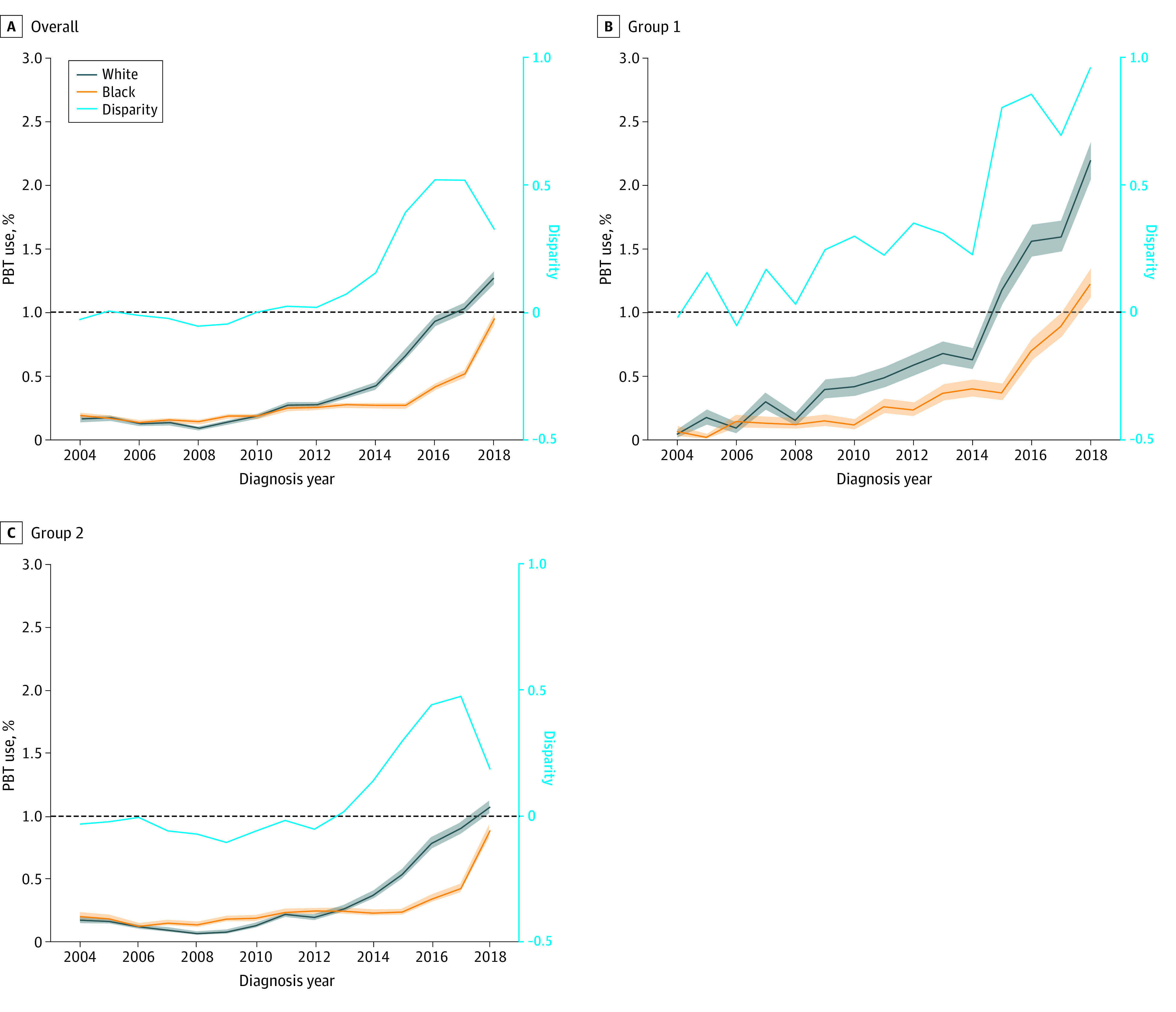
Overall Use of Proton Beam Therapy (PBT) and Use by American Society for Radiation Oncology Indication Group by Race, National Cancer Database (2004-2018) For each cancer site, Black and White patients were propensity score matched on age, sex, cancer stage at diagnosis, comorbidities, year of diagnosis, and geographic region. Disparity was calculated as the absolute difference between the rate of PBT use between Black and White patients. The dashed line at 0 represents no disparity.

**Figure 2.**
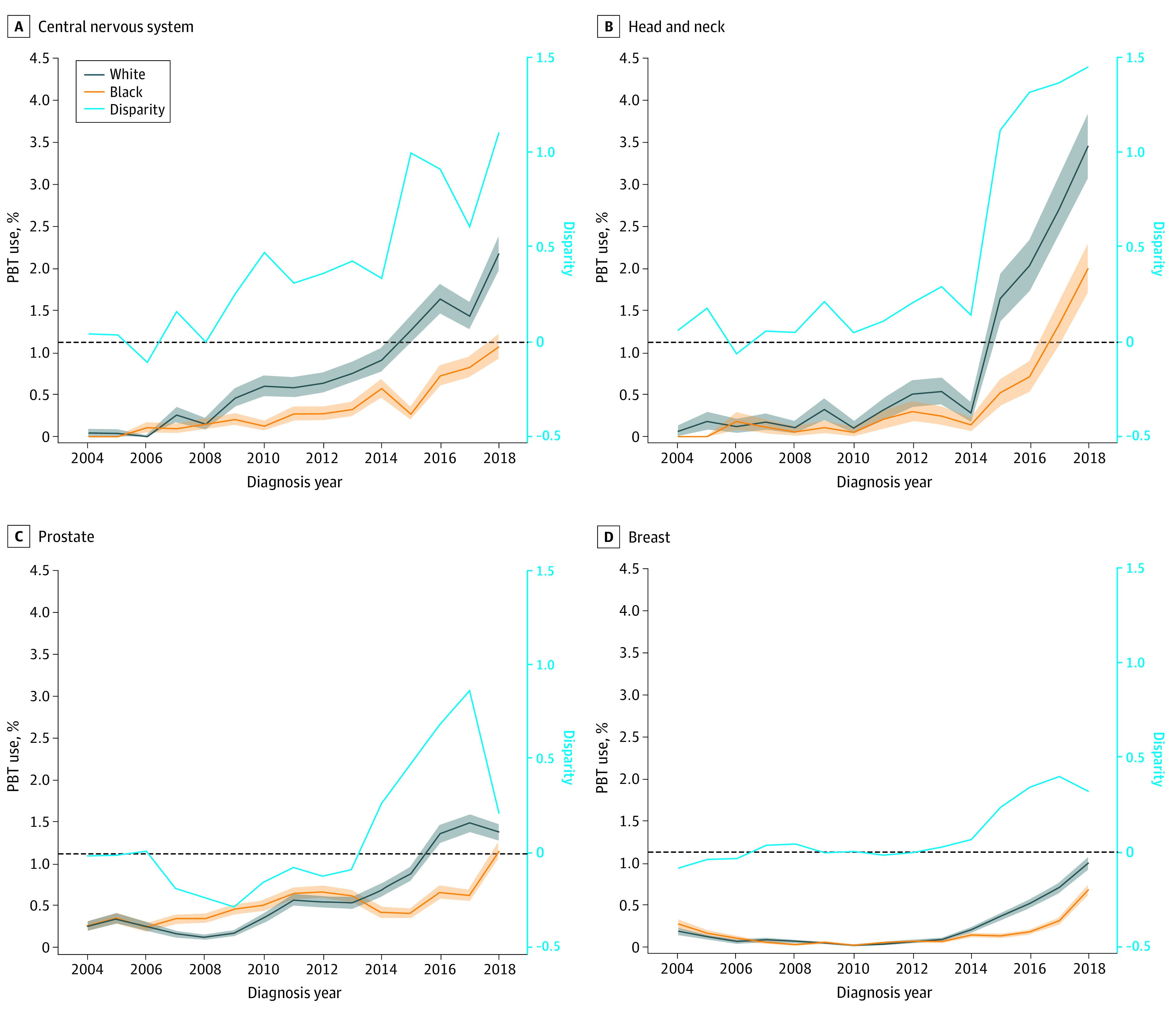
Use of Proton Beam Therapy (PBT) for the Group 1 and Group 2 Cancers Most Commonly Treated With PBT by Race, National Cancer Database (2004-2018) For each cancer site, Black and White patients were propensity score matched on age, sex, cancer stage at diagnosis, comorbidities, year of diagnosis, and geographic region. Disparity was calculated as the absolute difference between the rate of PBT use between Black and White patients. The dashed line at 0 represents no disparity.

The overall disparity measured as absolute difference in receipt of PBT between Black and White patients was statistically significant between 2010 and 2018 (APC = 0.07, *P* < .001) (Figure 1A). Racial disparities increased over time for group 1 (APC = 0.09, *P* < .001) and group 2 (APC = 0.06, *P* = .004) cancers. In group 1 cancers, disparities were greatest in 2018 (Figure 1B), whereas disparities for group 2 cancers decreased in 2018 (Figure 1C), mainly because of an increase in receipt of PBT among Black patients with prostate cancer (Figure 2).

Racial disparities narrowed but remained statistically significant after further matching on health insurance (OR, 0.72; 95% CI, 0.68-0.76) or income (OR, 0.73; 95% CI, 0.69-0.78) overall, by ASTRO group, and by all but 2 (hepatocellular and breast when matching on income) cancer sites (Table 2).

In sensitivity analyses, excluding stage IV cancers did not change the disparity estimates (eTable 5 in the Supplement), and disparity estimates were similar by breast cancer laterality (eTable 6 in the Supplement).

## Discussion

In this large, comprehensive, national evaluation of racial disparities in PBT receipt, Black patients were less likely to be treated with PBT than White patients with similar PBT eligibility and availability at diagnosis. Racial disparities were greater for group 1 cancers, for which PBT is the recommended RT modality, than for group 2 cancers.^43,54,55^ In addition, increase in availability of PBT during the study period coincided with increases rather than decreases in the racial disparity in PBT receipt for group 1 cancers, which was greatest in 2018. Further matching on health insurance or income narrowed but did not eliminate the racial disparity in receipt of PBT. These findings underscore the importance of identifying modifiable determinants of access to care other than regional availability to eliminate disparities in PBT receipt.

It is noteworthy that racial disparities in receipt of PBT were highest in group 1 cancers (which are rare and therefore require more frequent interactions with the health care system,^56,57^ thus increasing the cumulative burden of exposure to racism^58^), especially for rhabdomyosarcoma, the most common pediatric soft-tissue sarcoma, and CNS cancer, the next most commonly diagnosed cancer in children.^59^ Because PBT reduces the integral dose to surrounding healthy tissue, reducing the risk of secondary malignant neoplasms and other long-term consequences of RT, PBT is especially beneficial in children, making the racial disparities especially concerning.^60,61,62,63,64,65,66,67,68,69,70,71,72,73^ These results align with and extend findings from previous studies^11,27^ that reported racial disparities in receipt of PBT among pediatric patients with CNS cancer, with Black pediatric patients with cancer being less likely to receive PBT than White patients in previous studies, even when both racialized groups resided in high-income neighborhoods^16^ or when both were enrolled in clinical trials.^74^

Black patients were also less likely to receive PBT for hepatocellular and head and neck cancers, for which PBT is the recommended RT modality (group 1).^43^ One previous study^13^ used older data and restricted the analysis to patients receiving RT (which can underestimate the disparity because Black patients are less likely to receive any type of RT^6,75,76^) and did not find significant racial disparities in receipt of PBT for head and neck cancer. Another study^30^ used older data, restricted the analysis to nonsurgical patients receiving PBT or stereotactic body RT, and found that Black patients were less likely to receive PBT for hepatocellular cancer treatment than White patients.

Among group 2 cancers, racial disparities in PBT receipt were significant for prostate and breast cancers, the group 2 cancers most frequently treated with PBT,^57^ as well as lung and esophagus cancers. Our results are similar to those of older studies that reported racial disparities in receipt of PBT for prostate^7,10,17,25,28^ and breast cancer.^17,26^ In prostate cancer, previous studies have shown that White men who reside in more affluent regions and are diagnosed with lower-risk prostate cancer (for which RT provides no survival advantage over active monitoring)^77^ are more likely to receive PBT,^7^ raising concerns about overtreatment. In breast cancer, PBT is thought to have a potentially lower risk of cardiac toxic effects compared with photon therapy,^78,79,80^ which is especially important in avoiding late adverse effects among younger patients treated with RT targeted to the left side after mastectomy.^26^ We found that Black women, who are more frequently diagnosed with breast cancer at younger ages than White women,^81,82^ were half as likely to receive PBT targeted to the left breast as their White counterparts. The racial disparity in receipt of PBT for group 2 cancers seems to have decreased in more recent years, mainly because of an increase in PBT receipt among Black patients with prostate or breast cancer.

As the number of facilities offering PBT in the US increased, improving regional availability of this novel technology, racial disparities in receipt of PBT also increased, especially among patients diagnosed with group 1 cancers, for which PBT is the recommended treatment modality. This result suggests that developing and increasing regional availability of new cancer treatment technologies without addressing structural determinants of access to care can exacerbate instead of ameliorate racial disparities in receipt of quality cancer care.

Health insurance coverage type,^83,84,85^ including inconsistent coverage of PBT among different insurance providers,^86,87,88,89^ is as an important factor that contributes to racial disparities in receipt of PBT. Because the US system of employment-based health insurance coverage interacts with discriminatory hiring practices,^48^ health insurance is an especially important factor that contributes to racial disparities in receipt of PBT among patients with group 1 cancers, who are more likely to be diagnosed with cancer before 65 years of age,^57^ when US residents become age-eligible for universal health insurance coverage through Medicare. Therefore, policies such as the Patient Protection and Affordable Care Act, with multiple provisions to expand health insurance coverage options, can potentially help address disparities in access to care.^90,91,92^

Another important factor that contributes to racial disparities in receipt of PBT is SES. Individuals who reside in high-income areas are more likely to be treated with PBT than individuals who reside in low-income areas in the US.^57^ Structural racism is a primary cause of racial differences in SES by limiting access to education, employment opportunities, and intergenerational transfer of wealth.^47^ Living in socioeconomic disadvantage creates barriers in access to quality cancer care because of multiple factors, including but not limited to inability to afford out-of-pocket costs of cancer treatment; transportation insecurity; and lack of paid sick leave, job security, and work schedule flexibility to attend numerous cancer treatment appointments.^93^

After further matching on health insurance coverage type or income, racial disparities in receipt of PBT narrowed but were not eliminated, suggesting that other factors may contribute to these disparities, such as practitioner referral patterns^10,25,28^; practitioner implicit bias,^10,28^ whereby practitioner treatment recommendations may be influenced by a patient’s race^94,95,96^; and patient experiences of discrimination while interacting with the health care system.^97^ Increased diversity and training among health care professionals could improve sensitivity to cultural contexts^98^ and increase support to strategies that foster systemic changes necessary for equitable access to care.^99^

Because the study period encompasses the time when several PBT trials were being conducted, access to clinical trial enrollment might also have contributed to racial disparities in receipt of PBT. Black patients are less likely to be enrolled in clinical trials,^100,101,102^ including pediatric oncology clinical trials.^103^ A previous study^38^ found that, in addition to socioeconomic barriers to participation, bias and stereotyping among health care professionals influence recruitment of participants for oncology trials. In fact, an earlier study^74^ demonstrated that Black pediatric patients were less likely to receive PBT than White patients even while enrolled in clinical trials, in which treatment is highly standardized, further undermining the ability of the health care system to demonstrate trustworthiness to individuals from communities targeted for marginalization. Therefore, racial disparities in access to PBT could be diminished by policies and incentives aimed at developing a more diverse and culturally competent oncology workforce.^3^

### Strengths and Limitations

Our study has several strengths. It has the largest sample of PBT recipients to date, representing 70% of all patients with newly diagnosed cancer in the US,^41^ including information on treatment received outside the reporting facilities, which improves generalizability of our findings. In addition, to our knowledge, this is the first study to include all patients eligible for PBT and the first study to evaluate disparities in access to PBT regardless of receipt of RT (which can lead to underestimation of the disparity because Black patients are less likely to receive any type of RT). This strategy led to the identification of racial disparities in receipt of PBT among patients diagnosed with rhabdomyosarcoma, lung, and esophageal cancers, which have not been previously reported. Furthermore, we implemented several approaches to account for geographic heterogeneity and increased PBT availability over time. First, we excluded patients whose reporting facility or treating radiation oncologist had not treated at least 5 patients with PBT throughout the 14-year study period. Second, to address changes in regional density of PBT centers and the increase in availability of PBT over time,^57,104^ we matched Black and White patients on geographic region and diagnosis year. These approaches allowed us to estimate racial disparities in receipt of PBT that are not due to eligibility or regional availability of PBT and are not influenced by downstream consequences of exposure to structural racism (such as SES and health insurance coverage type). Third, we evaluated the contribution of modifiable factors, such as income and health insurance, to the racial disparities in PBT receipt.

Our study also has several limitations. First, propensity score methods are limited by their inability to control for unmeasured confounders.^105,106^ Second, the NCDB is not population based; therefore, patterns of PBT receipt might not be representative of the US population. However, NCDB facilities collect RT information even when provided at another facility. In our study, 40% of the patients who received PBT were treated somewhere other than the reporting facility, strengthening the generalizability of our findings. In addition, the demographic and clinical characteristics of patients with cancer in the NCDB are comparable to those from population-based cancer registries.^41^ Third, NCDB only collects information for first-course treatment. Therefore, no information is available on use of PBT for reirradiation or treatment of recurrent tumors.^107^ No information is available on social services provided at the facility level that might affect racial disparities in access to care (such as transportation services). Fourth, we were not able to look at disparities among patients of other racialized groups or ethnicities because of the small sample size.

## Conclusions

The findings of this cross-sectional study raise concerns regarding racial disparities in access to PBT and have policy importance. Racial disparities were greatest for cancers for which PBT is the recommended RT modality (group 1). Of note, racial disparity in PBT receipt did not decrease as the number of facilities that offer PBT in the US increased. The greatest racial disparity for group 1 was in 2018, the most recent year of available data. Further adjusting for modifiable factors known to contribute to racial disparities in access to quality cancer care (income and health insurance coverage type) narrowed but did not eliminate racial disparities in receipt of PBT. Future research should investigate the contribution of practitioner, facility, and health care system characteristics (such as referral patterns and reimbursement policies) to the racial disparity in receipt of PBT.

## Notes

Supplement. **eTable 1.** Standardized Differences Between Black and White Patients After Matching on PBT Eligibility and Availability, NCDB (2004-2018) **eTable 2.** Standardized Differences Between Black and White Patients After Matching on PBT Eligibility and Availability as Well as Health Insurance Coverage Type, NCDB (2004-2018) **eTable 3.** Standardized Differences Between Black and White Patients After Matching on PBT Eligibility and Availability as Well as Patient Residence Zip-Code Median Income Quintile, NCDB (2004-2018) **eTable 4.** Receipt of Proton Beam Therapy Among Black and White Patients, NCDB (2004-2018) **eTable 5.** Receipt of Proton Beam Therapy Among Black and White Patients Propensity Score Matched on PBT Eligibility and Availability Excluding Patients Diagnosed With Stage IV Cancer, NCDB (2004-2018) **eTable 6.** Receipt of Proton Beam Therapy Among Propensity Score Matched Black and White Patients by Breast Cancer Laterality, NCDB (2004-2018)
