# Supplementary material for: Association of Race With Receipt of Proton Beam Therapy for Patients With Newly Diagnosed Cancer in the US, 2004-2018
Source: JAMA Netw Open. 2022 Apr 26;5(4):e228970. doi: 10.1001/jamanetworkopen.2022.8970 (PMC9044116; doi:10.1001/jamanetworkopen.2022.8970)
Supplement: Supplement. — eTable 1. Standardized Differences Between Black and White Patients After Matching on PBT Eligibility and Availability, NCDB (2004-2018) eTable 2. Standardized Differences Between Black and White Patients After Matching on PBT Eligibility and Availability as Well as Health Insurance Coverage Type, NCDB (2004-2018) eTable 3. Standardized Differences Between Black and White Patients After Matching on PBT Eligibility and Availability as Well as Patient Residence Zip-Code Median Income Quintile, NCDB (2004-2018) eTable 4. Receipt of Proton Beam Therapy Among Black and White Patients, NCDB (2004-2018) eTable 5. Receipt of Proton Beam Therapy Among Black and White Patients Propensity Score Matched on PBT Eligibility and Availability Excluding Patients Diagnosed With Stage IV Cancer, NCDB (2004-2018) eTable 6. Receipt of Proton Beam Therapy Among Propensity Score Matched Black and White Patients by Breast Cancer Laterality, NCDB (2004-2018) [file jamanetwopen-e228970-s001.pdf]

Supplementary Online Content

Nogueira LM, Sineshaw HM, Jemal A, Pollack CE, Efstathiou JA, Yabroff KR. Association of race with receipt of proton beam therapy for patients with newly diagnosed cancer in the US, 2004-2018. *JAMA Netw Open*. 2022;5(4):e228970. doi:10.1001/jamanetworkopen.2022.8970

eTable 1. Standardized Differences Between Black and White Patients After Matching on PBT Eligibility and Availability, NCDB (2004-2018)

eTable 2. Standardized Differences Between Black and White Patients After Matching on PBT Eligibility and Availability as Well as Health Insurance Coverage Type, NCDB (2004-2018)

eTable 3. Standardized Differences Between Black and White Patients After Matching on PBT Eligibility and Availability as Well as Patient Residence Zip-Code Median Income Quintile, NCDB (2004-2018)

eTable 4. Receipt of Proton Beam Therapy Among Black and White Patients, NCDB (2004-2018)

eTable 5. Receipt of Proton Beam Therapy Among Black and White Patients Propensity Score Matched on PBT Eligibility and Availability Excluding Patients Diagnosed With Stage IV Cancer, NCDB (2004-2018)

eTable 6. Receipt of Proton Beam Therapy Among Propensity Score Matched Black and White Patients by Breast Cancer Laterality, NCDB (2004-2018)

This supplementary material has been provided by the authors to give readers additional information about their work.

© 2022 Nogueira LM et al. *JAMA Network Open.*

eTable 1. Standardized differences between Black and White patients after matching, NCDB (2004-2018)

Variable	White N (%)	Black N (%)	Standard Difference
Age, Mean (SD)	60.5 (13.3)	60.5 (13.3)	0.001
**Age**			
Children (<15)	3,672 (0.5)	3,727 (0.5)	0.001
AYA (15-39)	35,097 (5.2)	35,225 (5.2)	0.001
Adult (40-64)	375,650 (55.3)	375,370 (55.2)	0.001
Older Adult (65-74)	172,311 (25.3)	172,188 (25.3)	0.000
Elderly (75+)	93,153 (13.7)	93,373 (13.7)	0.001
**Sex**			
Male	312,430 (46.0)	311,377 (45.8)	0.003
Female	367,453 (54.0)	368,506 (54.2)	0.003
**PBT Group**			
Group 1	117,364 (17.3)	117,364 (17.3)	0.000
Group 2	562,519 (82.7)	562,519 (82.7)	0.000
**Cancer Site**			
Group 1			
Head & Neck	29,069 (4.3)	29,069 (4.3)	0.000
CNS	60,816 (8.9)	60,816 (8.9)	0.000
Hepatocellular	25,969 (3.8)	25,969 (3.8)	0.000
Skull and Spine	444 (0.1)	444 (0.1)	0.000
Ocular	535 (0.1)	535 (0.1)	0.000
Rhabdomyosarcoma	531 (0.1)	531 (0.1)	0.000
Group 2			
Prostate	170,435 (25.1)	170,435 (25.1)	0.000
Lung	91,479 (13.5)	91,479 (13.5)	0.000
Breast	222,036 (32.7)	222,036 (32.7)	0.000
Colon & Rectum	26,531 (3.9)	26,531 (3.9)	0.000
Anal	2,988 (0.4)	2,988 (0.4)	0.000
Uterus	10,547 (1.6)	10,547 (1.6)	0.000
Cervix	10,479 (1.5)	10,479 (1.5)	0.000
Pancreas	15,540 (2.3)	15,540 (2.3)	0.000
Esophagus	7,185 (1.1)	7,185 (1.1)	0.000
Hodgkin Lymphoma	5,299 (0.8)	5,299 (0.8)	0.000
**Stage**			
Stage 0	44,134 (6.5)	44,245 (6.5)	0.001
Stage I	154,489 (22.7)	153,953 (22.6)	0.002
Stage II	207,802 (30.6)	208,152 (30.6)	0.001
Stage III	99,968 (14.7)	99,382 (14.6)	0.002
Stage IV	83,657 (12.3)	83,288 (12.3)	0.002
Unknown	89,833 (13.2)	90,863 (13.4)	0.004
**Region**			
Northeast	124,438 (18.3)	126,189 (18.6)	0.007

© 2022 Nogueira LM et al. *JAMA Network Open.*

Midwest	129,276 (19.0)	130,136 (19.1)	0.003
South	388,454 (57.1)	385,337 (56.7)	0.009
West	37,715 (5.5)	38,221 (5.6)	0.003
**Comorbidity**			
0	499,774 (73.5)	493,933 (72.6)	0.019
1	122,785 (18.1)	124,749 (18.3)	0.007
2+	57,324 (8.4)	61,201 (9.0)	0.020
**Diagnosis Year**			
2004	31,872 (4.7)	31,915 (4.7)	0.000
2005	33,500 (4.9)	33,464 (4.9)	0.000
2006	36,361 (5.3)	36,326 (5.3)	0.000
2007	39,436 (5.8)	39,430 (5.8)	0.000
2008	41,090 (6.0)	41,253 (6.1)	0.001
2009	44,140 (6.5)	44,162 (6.5)	0.000
2010	44,633 (6.6)	44,751 (6.6)	0.001
2011	46,753 (6.9)	46,691 (6.9)	0.000
2012	47,226 (6.9)	47,272 (7.0)	0.000
2013	48,983 (7.2)	48,985 (7.2)	0.000
2014	49,992 (7.4)	50,036 (7.4)	0.000
2015	51,355 (7.6)	51,414 (7.6)	0.000
2016	52,949 (7.8)	52,678 (7.7)	0.001
2017	55,161 (8.1)	55,005 (8.1)	0.001
2018	56,432 (8.3)	56,501 (8.3)	0.000

[a] Standard difference represents the difference in means between the two groups in units of standard deviation, so it does not depend on the unit of measurement. Standard difference ≥.2 indicates imbalance between the groups.
Notes: AYA = Adolescent and young Adult, CNS = Central Nervous System,

© 2022 Nogueira LM et al. *JAMA Network Open.*

eTable 2. Standardized differences between Black and White patients after matching on PBT eligibility and availability as well as health insurance coverage type, NCDB (2004-2018)

Variable	White N (%)	Black N (%)	Standard Difference [a]
**Age**			
Children (<15)	3,725 (0.5)	3,760 (0.5)	0.0007
AYA (15-39)	33,048 (4.8)	33,486 (4.9)	0.0030
Adult (40-64)	376,479 (55.0)	375,790 (54.9)	0.0020
Older Adult (65-74)	177,549 (26.0)	177,180 (25.9)	0.0012
Elderly (75+)	93,175 (13.6)	93,760 (13.7)	0.0025
**Gender**			
Male	317,997 (46.5)	317,361 (46.4)	0.0019
Female	365,979 (53.5)	366,615 (53.6)	0.0019
**PBT Group**			
Group 1	117,623 (17.2)	117,623 (17.2)	0.0000
Group 2	566,353 (82.8)	566,353 (82.8)	0.0000
**Cancer Site**			
Group 1			
Head & Neck	30,314 (4.4)	30,314 (4.4)	0.0000
CNS	59,397 (8.7)	59,397 (8.7)	0.0000
Hepatocellular	26,310 (3.8)	26,310 (3.8)	0.0000
Skull and Spine	416 (0.1)	416 (0.1)	0.0000
Ocular	623 (0.1)	623 (0.1)	0.0000
Rhabdomyosarcoma	563 (0.1)	563 (0.1)	0.0000
Group 2			
Prostate	163,435 (23.9)	163,435 (23.9)	0.0000
Lung	109,810 (16.1)	109,810 (16.1)	0.0000
Breast	215,114 (31.5)	215,114 (31.5)	0.0000
Colon & Rectum	26,804 (3.9)	26,804 (3.9)	0.0000
Anal	2,951 (0.4)	2,951 (0.4)	0.0000
Uterus	10,331 (1.5)	10,331 (1.5)	0.0000
Cervix	10,251 (1.5)	10,251 (1.5)	0.0000
Pancreas	15,402 (2.3)	15,402 (2.3)	0.0000
Esophagus	7,027 (1.0)	7,027 (1.0)	0.0000
Hodgkin Lymphoma	5,228 (0.8)	5,228 (0.8)	0.0000
**Stage**			
Stage 0	42,871 (6.3)	42,988 (6.3)	0.0007
Stage I	155,240 (22.7)	154,946 (22.7)	0.0010
Stage II	201,604 (29.5)	201,872 (29.5)	0.0009
Stage III	103,282 (15.1)	103,299 (15.1)	0.0001
Stage IV	93,500 (13.7)	92,497 (13.5)	0.0043
Unknown	87,479 (12.8)	88,374 (12.9)	0.0039
**Region**			
Northeast	120,188 (17.6)	123,012 (18.0)	0.0108

© 2022 Nogueira LM et al. *JAMA Network Open.*

Midwest	127,403 (18.6)	128,702 (18.8)	0.0049
South	399,278 (58.4)	394,307 (57.6)	0.0147
West	37,107 (5.4)	37,955 (5.5)	0.0054
**Comorbidity**			
0	500,049 (73.1)	497,310 (72.7)	0.0090
1	124,493 (18.2)	125,214 (18.3)	0.0027
2+	59,434 (8.7)	61,452 (9.0)	0.0104
**Diagnosis Year**			
2004	31,356 (4.6)	31,533 (4.6)	0.0012
2005	33,195 (4.9)	33,270 (4.9)	0.0005
2006	36,024 (5.3)	36,055 (5.3)	0.0002
2007	39,092 (5.7)	39,312 (5.7)	0.0014
2008	41,229 (6.0)	41,381 (6.1)	0.0009
2009	44,178 (6.5)	44,285 (6.5)	0.0006
2010	44,582 (6.5)	44,772 (6.5)	0.0011
2011	46,670 (6.8)	46,783 (6.8)	0.0007
2012	47,179 (6.9)	47,431 (6.9)	0.0015
2013	49,575 (7.2)	49,534 (7.2)	0.0002
2014	50,736 (7.4)	50,719 (7.4)	0.0001
2015	52,602 (7.7)	51,920 (7.6)	0.0038
2016	54,098 (7.9)	53,561 (7.8)	0.0029
2017	56,606 (8.3)	56,159 (8.2)	0.0024
2018	56,854 (8.3)	57,261 (8.4)	0.0022
**Insurance**			
Private	297,864 (43.5)	294,029 (43.0)	0.0113
Uninsured	30,843 (4.5)	31,497 (4.6)	0.0046
Medicaid	78,664 (11.5)	80,336 (11.7)	0.0076
Medicare	268,846 (39.3)	270,016 (39.5)	0.0035
Other	7,759 (1.1)	8,098 (1.2)	0.0046

[a] Standard difference represents the difference in means between the two groups in units of standard deviation, so it does not depend on the unit of measurement. Standard difference ≥.2 indicates imbalance between the groups.
Notes: AYA = Adolescent and young Adult, CNS = Central Nervous System

© 2022 Nogueira LM et al. *JAMA Network Open.*

eTable 3. Standardized differences between Black and White patients after matching on PBT eligibility and availability as well as patient residence zip-code median income quintile, NCDB (2004-2018)

Variable	White N (%)	Black N (%)	Standard Difference [a]
**Age**			
Children (<15)	3,726 (0.5)	3,832 (0.6)	0.0021
AYA (15-39)	31,904 (4.7)	32,727 (4.8)	0.0057
Adult (40-64)	375,008 (54.9)	374,706 (54.9)	0.0009
Older Adult (65-74)	178,947 (26.2)	177,615 (26.0)	0.0044
Elderly (75+)	92,904 (13.6)	93,609 (13.7)	0.0030
**Gender**			
Male	320,366 (46.9)	318,480 (46.7)	0.0055
Female	362,123 (53.1)	364,009 (53.3)	0.0055
**PBT Group**			
Group 1	119,739 (17.5)	119,739 (17.5)	0.0000
Group 2	562,750 (82.5)	562,750 (82.5)	0.0000
**Cancer Site**			
Group 1			
Head & Neck	30,894 (4.5)	30,894 (4.5)	0.0000
CNS	60,575 (8.9)	60,575 (8.9)	0.0000
Hepatocellular	26,652 (3.9)	26,652 (3.9)	0.0000
Skull and Spine	432 (0.1)	432 (0.1)	0.0000
Ocular	626 (0.1)	626 (0.1)	0.0000
Rhabdomyosarcoma	560 (0.1)	560 (0.1)	0.0000
Group 2			
Prostate	163,685 (24.0)	163,685 (24.0)	0.0000
Lung	111,568 (16.3)	111,568 (16.3)	0.0000
Breast	212,936 (31.2)	212,936 (31.2)	0.0000
Colon & Rectum	25,597 (3.8)	25,597 (3.8)	0.0000
Anal	2,235 (0.3)	2,235 (0.3)	0.0000
Uterus	10,285 (1.5)	10,285 (1.5)	0.0000
Cervix	9,315 (1.4)	9,315 (1.4)	0.0000
Pancreas	14,760 (2.2)	14,760 (2.2)	0.0000
Esophagus	7,094 (1.0)	7,094 (1.0)	0.0000
Hodgkin Lymphoma	5,275 (0.8)	5,275 (0.8)	0.0000
**Stage**			
Stage 0	42,272 (6.2)	42,344 (6.2)	0.0004
Stage I	157,033 (23.0)	155,168 (22.7)	0.0065
Stage II	200,555 (29.4)	201,038 (29.5)	0.0016
Stage III	101,816 (14.9)	102,162 (15.0)	0.0014
Stage IV	92,599 (13.6)	92,519 (13.6)	0.0003
Unknown	88,214 (12.9)	89,258 (13.1)	0.0045
**Region**			
Northeast	113,609 (16.6)	120,988 (17.7)	0.0287

© 2022 Nogueira LM et al. *JAMA Network Open.*

Midwest	121,317 (17.8)	126,222 (18.5)	0.0187
South	410,108 (60.1)	397,345 (58.2)	0.0381
West	37,455 (5.5)	37,934 (5.6)	0.0031
**Comorbidity**			
0	505,431 (74.1)	499,062 (73.1)	0.0212
1	120,689 (17.7)	123,324 (18.1)	0.0101
2+	56,369 (8.3)	60,103 (8.8)	0.0196
**Diagnosis Year**			
2004	32,100 (4.7)	31,462 (4.6)	0.0044
2005	33,307 (4.9)	33,158 (4.9)	0.0010
2006	36,146 (5.3)	36,079 (5.3)	0.0004
2007	38,719 (5.7)	39,112 (5.7)	0.0025
2008	40,940 (6.0)	41,255 (6.0)	0.0019
2009	43,868 (6.4)	44,197 (6.5)	0.0020
2010	43,958 (6.4)	44,807 (6.6)	0.0050
2011	46,411 (6.8)	46,830 (6.9)	0.0024
2012	46,813 (6.9)	47,580 (7.0)	0.0044
2013	49,018 (7.2)	49,643 (7.3)	0.0035
2014	50,391 (7.4)	50,603 (7.4)	0.0012
2015	52,686 (7.7)	52,086 (7.6)	0.0033
2016	54,100 (7.9)	53,204 (7.8)	0.0049
2017	56,860 (8.3)	55,715 (8.2)	0.0061
2018	57,172 (8.4)	56,758 (8.3)	0.0022
**Income**			
<$36,000	225,212 (33.0)	229,278 (33.6)	0.0126
$36,000-$43,999	135,295 (19.8)	131,294 (19.2)	0.0148
$44,000-$52,999	110,745 (16.2)	108,253 (15.9)	0.0099
$53,000-$68,999	120,447 (17.6)	119,972 (17.6)	0.0018
$69,000+	90,790 (13.3)	93,692 (13.7)	0.0124

[a] Standard difference represents the difference in means between the two groups in units of standard deviation, so it does not depend on the unit of measurement. Standard difference ≥.2 indicates imbalance between the groups.
Notes: AYA = Adolescent and young Adult, CNS = Central Nervous System

© 2022 Nogueira LM et al. *JAMA Network Open.*

eTable 4. Receipt of Proton Beam Therapy among Black and White patients, NCDB (2004-2018)

	No PBT N (%)	PBT N (%)	Crude OR (95% CI)
**Overall**			
NH White	4,492,055 (99.5)	23,624 (0.5)	
NH Black	708,081 (99.7)	2,169 (0.3)	0.58 (0.56, 0.61)
**ASTRO Indication Group**			
**Group 1**			
NH White	769,158 (99.1)	7,010 (0.9)	
NH Black	120,588 (99.6)	494 (0.4)	0.45 (0.41, 0.49)
**Group 2**			
NH White	3,722,897 (99.6)	16,614 (0.4)	
NH Black	587,493 (99.7)	1,675 (0.3)	0.64 (0.61, 0.67)
**Cancer Sites**			
**Group 1**			
Head & Neck			
NH White	221,697 (99.2)	1,743 (0.8)	
NH Black	31,081 (99.6)	133 (0.4)	0.54 (0.46, 0.65)
CNS			
NH White	400,039 (99.3)	2,766 (0.7)	
NH Black	60,860 (99.6)	244 (0.4)	0.58 (0.51, 0.66)
Hepatocellular			
NH White	120,293 (99.8)	206 (0.2)	
NH Black	27,019 (99.9)	21 (0.1)	0.45 (0.29, 0.71)
Skull & Spine			
NH White	4,230 (89.2)	512 (10.8)	
NH Black	419 (92.1)	36 (7.9)	0.71 (0.50, 1.01)
Ocular			
NH White	20,881 (93.5)	1,460 (6.5)	
NH Black	650 (98.2)	12 (1.8)	0.26 (0.15, 0.47)
Rhabdomyosarcoma			
NH White	2,018 (86.2)	323 (13.8)	
NH Black	559 (92.1)	48 (7.9)	0.54 (0.39, 0.74)
**Group 2**			
Prostate			
NH White	897,492 (98.9)	10,386 (1.1)	
NH Black	170,634 (99.5)	911 (0.5)	0.46 (0.43, 0.49)
Lung			
NH White	829,214 (99.8)	1,557 (0.2)	
NH Black	111,904 (99.8)	208 (0.2)	0.99 (0.86, 1.14)
Breast			
NH White	1,459,492 (99.8)	3,113 (0.2)	
NH Black	224,878 (99.8)	358 (0.2)	0.75 (0.67, 0.83)
Esophagus			
NH White	79,680 (99.3)	530 (0.7)	
NH Black	7,215 (99.6)	29 (0.4)	0.60 (0.42, 0.88)
Hodgkin Lymphoma			
NH White	32,629 (99.2)	269 (0.8)	
NH Black	5,423 (99.3)	37 (0.7)	0.83 (0.59, 1.17)
Colorectal			
NH White	209,354 (99.9)	279 (0.1)	
NH Black	27,429 (99.9)	37 (0.1)	1.01 (0.72, 1.43)
Anal			
NH White	20,627 (99.4)	129 (0.6)	
NH Black	3,142 (99.3)	21 (0.7)	1.07 (0.67, 1.70)
Pancreas			
NH White	105,493 (99.8)	241 (0.2)	
NH Black	15,730 (99.8)	32 (0.2)	0.89 (0.62, 1.29)
Cervix			
NH White	40,414 (99.8)	68 (0.2)	
NH Black	10,538 (99.8)	23 (0.2)	1.30 (0.81, 2.08)

© 2022 Nogueira LM et al. *JAMA Network Open.*

eTable 5. Receipt of Proton Beam Therapy among Black and White patients propensity score matched on eligibility and availability excluding patients diagnosed with stage IV cancer, NCDB (2004-2018)

	No PBT N (%)	PBT N (%)	Odds Ratios (95% CI)
**Group 1**			
Hepatocellular			
NH White	16,973 (99.8)	29 (0.2)	
NH Black	16,942 (99.9)	17 (0.1)	0.59 (0.32, 1.07)
Skull & Spine			
NH White	366 (90.8)	37 (9.2)	
NH Black	373 (91.6)	34 (8.4)	0.90 (0.55, 1.47)
Ocular			
NH White	588 (99.2)	5 (0.8)	
NH Black	580 (98.0)	12 (2.0)	2.43 (0.85, 6.95)
Rhabdomyosarcoma			
NH White	376 (83.7)	73 (16.3)	
NH Black	423 (93.4)	30 (6.6)	0.37 (0.23, 0.57)
**Group 2**			
Prostate			
NH White	147,861 (99.2)	1,241 (0.8)	
NH Black	147,142 (99.4)	878 (0.6)	0.71 (0.65, 0.78)
Breast			
NH White	169,235 (99.8)	374 (0.2)	
NH Black	169,369 (99.9)	240 (0.1)	0.64 (0.55, 0.75)
Lung			
NH White	64,416 (99.7)	197 (0.3)	
NH Black	64,345 (99.7)	184 (0.3)	0.94 (0.76, 1.14)
Esophagus			
NH White	4,464 (99.2)	35 (0.8)	
NH Black	4,481 (99.4)	26 (0.6)	0.74 (0.44, 1.23)
Hodgkin Lymphoma			
NH White	3,754 (99.1)	33 (0.9)	
NH Black	3,725 (99.2)	29 (0.8)	0.89 (0.54, 1.46)
Colorectal			
NH White	18,333 (99.9)	23 (0.1)	
NH Black	18,336 (99.8)	34 (0.2)	1.48 (0.87, 2.51)
Anal			
NH White	2,761 (99.6)	12 (0.4)	
NH Black	2,754 (99.3)	19 (0.7)	1.59 (0.77, 3.28)
Pancreas			
NH White	12,458 (99.8)	25 (0.2)	
NH Black	12,452 (99.8)	31 (0.2)	1.24 (0.73, 2.10)
Cervix			
NH White	8,278 (99.9)	10 (0.1)	
NH Black	8,272 (99.8)	20 (0.2)	2.00 (0.94, 4.28)
Uterus			
NH White	9,149 (99.9)	11 (0.1)	
NH Black	9,125 (99.8)	17 (0.2)	1.55 (0.73, 3.31)

© 2022 Nogueira LM et al. *JAMA Network Open.*

eTable 6. Receipt of Proton Beam Therapy among propensity score matched Black and White patients by breast cancer laterality, NCDB (2004-2018)

Laterality	No PBT N (%)	PBT N (%)	Crude Odds Ratios (95% CI)
**Right**			
NH White	109,062 (99.8)	240 (0.2)	
NH Black	109,476 (99.8)	172 (0.2)	0.71 (0.59, 0.87)
**Left**			
NH White	111,962 (99.7)	340 (0.3)	
NH Black	111,870 (99.8)	175 (0.2)	0.52 (0.43, 0.62)

© 2022 Nogueira LM et al. *JAMA Network Open.*
